# One-Year Survival of Ischemic Stroke Patients Requiring Mechanical Ventilation

**DOI:** 10.1007/s12028-023-01674-9

**Published:** 2023-02-09

**Authors:** Harri Isokuortti, Jyri J. Virta, Sami Curtze, Marjaana Tiainen

**Affiliations:** 1 Department of Neurosurgery, Helsinki University Hospital, University of Helsinki, Helsinki, Finland; 2 Division of Intensive Care Medicine, Department of Anaesthesiology, Intensive Care and Pain Medicine, Helsinki University Hospital, University of Helsinki, Helsinki, Finland; 3 Department of Neurology, Helsinki University Hospital, University of Helsinki, Helsinki, Finland

**Keywords:** Neurointensive care, Mechanical ventilation, Ischemic stroke, Mortality, Functional outcome

## Abstract

**Background:**

The outcome of patients with acute ischemic stroke who require mechanical ventilation has been poor. Intubation due to a reversible condition could be associated with better 1-year survival.

**Methods:**

All adult patients treated in Helsinki University Hospital in 2016–2020 who were admitted because of an ischemic stroke (either stroke or thrombosis seen on imaging) and needed mechanical ventilation were included in this retrospective cohort study. Data on demographics, medical history, index stroke, and indication for intubation were collected. The primary outcome was 1-year mortality. Secondary outcomes were modified Rankin Scale (mRS) score at 3 months and living arrangements at 1 year.

**Results:**

The mean age of the cohort (*N* = 121) was 66 ± 11 (mean ± SD) years, and the mean admission National Institutes of Health Stroke Scale score was 17 ± 10. Forty-four (36%) patients were male. The most common indication for intubation was unconsciousness (51%), followed by respiratory failure or airway compromise (28%). One-year mortality was 55%. Three-month mRS scores were available for 114 (94%) patients, with the following distribution: 0–2, 18%; 3–5, 28%; and 6 (dead), 54%. Of the 1-year survivors, 72% were living at home. In the multivariate analysis, only age over 75 years and intubation due to unconsciousness, respiratory failure, or cardiac arrest remained significantly associated with mortality.

**Conclusions:**

The indication for intubation seems to significantly affect outcome. Functional outcome at 3 months is often poor, but a great majority of 1-year survivors are able to live at home.

**Supplementary Information:**

The online version contains supplementary material available at 10.1007/s12028-023-01674-9.

## Introduction

Patients with acute ischemic stroke may require mechanical ventilation for various reasons, including unconsciousness, severe agitation, seizures, respiratory failure, and procedural sedation [1–3]. According to a previous study, almost 10% of patients with ischemic stroke require mechanical ventilation [4], and mechanical ventilation appears to be a major predictor of mortality [5]. Mechanically ventilated patients have poor prognosis, with hospital and 1-year mortalities of 55–57% [4, 6–8] and 60–92% [9–13], respectively. Similarly, in a large population-based study of 798,255 patients with acute stroke, need for mechanical ventilation reduced the fraction of patients discharged to home from 37 to 12% [4].

Mechanical ventilation may be required for reversible conditions (e.g., status epilepticus, pneumonia, sepsis, or agitation) that may be associated with more favorable outcome [14]. Hence, need for mechanical ventilation should not be seen merely as a marker of stroke severity. Studies evaluating predictors of outcome in mechanically ventilated patients with ischemic stroke have shown that age, impaired consciousness, absent brainstem reflexes, and infarct volume are associated with impaired survival [6, 9–11, 13, 15, 16]. Most of these studies were done before the year 2000, but neurocritical care and acute stroke care have significantly evolved since then [17]. Studies on long-term outcome of patients with ischemic stroke who require mechanical ventilation are scarce [8].

We aimed to study the association of indication for intubation with 1-year survival in patients with acute ischemic stroke who require mechanical ventilation. We hypothesized that intubation due to a possibly reversible condition would be associated with better 1-year survival.

## Methods

### Study Setting and Population

We conducted a single-center retrospective cohort study of patients with ischemic stroke who required mechanical ventilation and were treated at Helsinki University Hospital between 2016 and 2020. Our facility is a comprehensive tertiary stroke center that covered an area of 1.9 million people in 2019. In Finland, all patients with acute ischemic stroke are treated in public hospitals.

To identify patients with ischemic stroke who required mechanical ventilation, we screened patients treated at an acute stroke care ward and two intensive care units (ICUs). The acute stroke care ward in our hospital is a high-dependency unit capable of treating patients requiring mechanical ventilation or invasive blood pressure measurement. All intubated patients with stroke are treated at these three units. For the two ICUs, we manually screened the electronic health records of all patients with an International *Statistical Classification of Diseases and Related Health Problems, Tenth Revision* (ICD-10) diagnosis code for ischemic stroke (i.e., I63.0–I63.9) to identify those requiring mechanical ventilation. For the acute stroke care ward, we obtained patients with an ICD-10 diagnosis code for ischemic stroke and a Nordic Classification of Surgical Procedures code for mechanical ventilation, intubation, or general anesthesia.

**Fig. 1:**
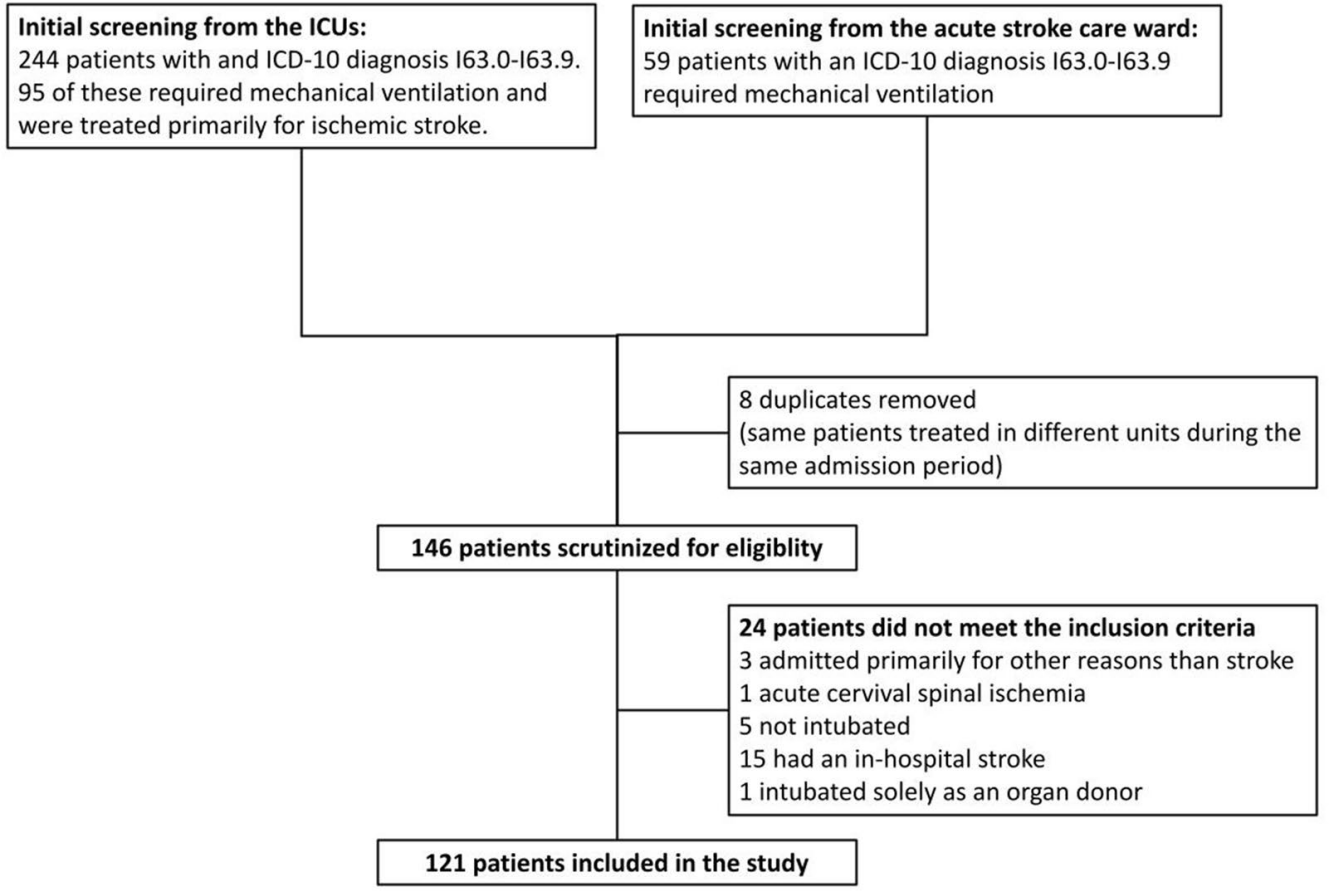
Flowchart illustrating data acquisition. ICU, intensive care unit, ICD-10, International Statistical Classification of Diseases and Related Health Problems, Tenth Revision

After initial screening, we scrutinized the patients’ electronic health records and imaging studies to identify patients who met the inclusion criteria. We included adult patients whose primary reason for admission was an ischemic stroke, patients who required mechanical ventilation at some point during their admission, and patients who had a verified infarction and/or cerebral artery thrombosis on imaging. We excluded patients who suffered a stroke while they were treated in the hospital (e.g., perioperative stroke); patients whose stroke was caused by septic emboli (i.e., endocarditis), meningitis, or sinus thrombosis; and patients who were admitted solely as potential organ donors. A study flowchart is shown in Fig. 1.

### Data Collection

After selecting the patients, we collected the following variables from electronic health records: age at admission; sex; Charlson Comorbidity Index (CCI) [18]; preadmission Clinical Frailty Scale score [19] (dichotomized as 1–4 indicating nonfrail and 5–9 indicating frail); history of hypertension, hypercholesterolemia, or diabetes (either an ICD-10 diagnosis or on medication at admission); and smoking status (current smoker, ex-smoker, never smoked).

For ischemic stroke, we obtained the National Institutes of Health Stroke Scale (NIHSS) [20] and Glasgow Coma Scale (GCS) [21] scores on admission. For patients intubated before admission at our hospital, we used the last recorded GCS score before intubation. We also recorded infarct location (anterior and/or posterior circulation) and possible acute stroke treatments (intravenous [IV] thrombolysis, thrombectomy, hemicraniectomy/other neurosurgical intervention).

Regarding mechanical ventilation, we recorded the time of intubation in relation to stroke onset, duration of mechanical ventilation, possible reintubation, tracheostomy, and withdrawal of care during mechanical ventilation. We classified the primary reason for intubation as follows: (1) unconsciousness, (2) active delirium or agitation, (3) respiratory failure or airway compromise, (4) hemodynamic instability, (5) epileptic seizure, (6) cardiac arrest, and (7) preprocedural (e.g., for thrombectomy). The decision to intubate was made by the treating intensive care physician. Unconscious patients were considered unarousable, unresponsive, and/or unable to protect their airway. The guidelines of our stroke center prefer procedural sedation to general anesthesia for thrombectomy.

During the chart review, the study authors HI, JJV, and MT assessed two thirds of the patients. After the initial chart review, the indications for intubation were cross-checked. Any discrepancies were then assessed by study author SC, who did not participate in the first round of chart review. As a final result, all patients were given a consensus decision about the indication for intubation.

### Outcomes

Our primary outcome was all-cause mortality at 1 year. We also collected mortality at the acute stroke care ward or ICU, in-hospital mortality, and modified Rankin Scale (mRS) [22] score at 3 months. For patients who underwent IV thrombolysis or thrombectomy, mRS scores were obtained from the previously described prospectively collected Helsinki Stroke Quality Registry [23]. For the rest of the patients, mRS scores were estimated retrospectively from electronic health records.

We also collected data on discharge destination (home, rehabilitation, other ward, or sheltered care), whether patients were discharged to home at some point after discharge from the university hospital, and residential status at 1 year (home with or without home care service, sheltered care, or ward).

### Ethical Considerations

The research board of the Neurocenter at Helsinki University Hospital granted the research permit for this registry study (HUS/190/2021). At our institution, this research permit is sufficient for a register study, and a separate ethical board review was not required at our institution. This study was conducted in accordance with the Declaration of Helsinki as revised in 2013.

### Statistical Analyses

We report mean values with standard deviations (SDs) for continuous variables and proportions for categorical variables. Risk factors for 1-year mortality and poor outcome at 3 months (defined as mRS scores 3–6) were assessed with univariate and multivariate logistic regression. In addition to the indication for intubation, the multivariate model included age, NIHSS score at admission [24, 25], and CCI [26, 27] because these have been associated with mortality in previous studies. Any acute stroke therapies (IV thrombolysis and/or endovascular thrombectomy) were also included because these have been shown to improve the outcome [28–30]. We report odds ratios (ORs) with 95% confidence intervals (CIs) for the regression analyses. Life tables and Kaplan–Meier survival curves were calculated for 1-year mortality. A *p* value < 0.05 was considered significant for all analyses. We used IBM SPSS Statistics for Macintosh (versions 25–27; IBM Corp, Armonk, NY) for statistical analyses.

## Results

### Patient and Stroke Characteristics

The initial screening of the ICUs resulted in 244 patients, of whom 95 required mechanical ventilation and were primarily treated for ischemic stroke. Likewise, the initial screening of the acute stroke care ward resulted in 59 patients. After removing eight duplicates (i.e., same patients treated in different units during the admission), 146 patients were scrutinized for eligibility, and, finally, 121 patients were included in the study (Fig. 1). There were 33 patients who were intubated outside of our institution. The reason for intubation was determined on the basis of the information in the referral to our institution. Interrater agreement on the reasons for intubation was 81% (Cohen’s *κ* 0.72).

Patient and stroke characteristics are shown in Table 1. Briefly, the mean age of the patients was 66 ± 11 (mean ± SD) years, 36% were men, and a history of hypertension or hypercholesterolemia was common. The mean admission NIHSS score was 17 ± 10. Half of the patients received IV thrombolysis and/or thrombectomy. Fourteen percent required a neurosurgical intervention.

**Table 1:** Characteristics of the 121 patients included in the study

Demographic information	Patients (*n* = 121)
Age (years), mean ± SD	66.3 ± 11.3
Under 65 years, *n* (%)	44 (36%)
65–75 years, *n* (%)	52 (43%)
Over 75 years, *n* (%)	25 (21%)
Male sex, *n* (%)	44 (36%)
History of hypertension, *n* (%)	71 (59%)
History of hypercholesterolemia, *n* (%)	49 (41%)
History of diabetes, *n* (%)	21 (17%)
Smoking history, *n* (%)	
Never smoked	37 (31%)
Ex-smoker	24 (20%)
Current smoker	32 (26%)
Unknown	28 (23%)
Charlson comorbidity index,^a^ *n* (%)	
0	62 (53%)
1 or 2	40 (34%)
At least 3	17 (14%)
Clinical frailty scale 1–4, *n* (%)	114 (94%)
Clinical frailty scale 5–9, *n* (%)	7 (6%)
NIHSS at admission,^b^ mean ± SD	17.4 ± 10.2
0–4, *n* (%)	15 (13%)
5–15, *n* (%)	36 (31%)
16–20, *n* (%)	22 (19%)
21–42, *n* (%)	44 (38%)
GCS^c^, *n* (%)	
3–8	21 (19%)
9–12	31 (29%)
13–15	56 (52%)
Infarct location, *n* (%)	
Anterior circulation	77 (64%)
Posterior circulation	40 (33%)
Both	4 (3%)
IV thrombolysis, *n* (%)	57 (47%)
Thrombectomy, *n* (%)	60 (50%)
Neurosurgical intervention, *n* (%)	17 (14%)
3-month mRS 0–2,^d^ *n* (%)	20 (18%)
3-month mRS 3–6,^d^ *n* (%)	94 (83%)
ICU mortality, *n* (%)	31 (26%)
12-month mortality, *n* (%)	66 (55%)

GCS, Glasgow Coma Scale, ICU, intensive care unit, IV, intravenous, mRS, modified Rankin Scale, NIHSS, National Institutes of Health Stroke Scale

^a^Missing for two patients

^b^Missing for four patients

^c^Missing for 13 patients

^d^Three-month mRS missing for seven patients

For those patients who died during their hospital stay, we received the information from the electronical health repository. In Finland, all of the deaths are registered by Statistics Finland. We were able to make an inquiry about the vital status of each patient from the Finnish Population Information System to ascertain the death of this person and to obtain the date of death when needed.

### Ventilator and ICU Treatment

The intubation indications are shown in Table 2. The most common indication was unconsciousness (51%), followed by respiratory failure or airway compromise (27%). One hundred two (84%) patients were intubated during the first 2 days following admission, and eight (7%) patients were intubated preprocedurally (i.e., before thrombectomy and/or thrombolysis). Patients with posterior or both anterior and posterior infarcts were more often intubated because of unconsciousness compared with patients with only anterior infarcts (66% vs. 43%, respectively; *χ*^2^ *p* = 0.015). However, infarct location was not associated with 1-year mortality (anterior infarction 56% and posterior infarction 52%; *χ*^2^ *p* = 0.70).

**Table 2:** Indications for intubation, 3-month mRS, and 1-year mortality stratified by indication

Reason for intubation	Number of patients (%)	mRS 3–6 at 3 months (%)^a^	Dead at 12 months (%)
Unconsciousness	62 (51%)	84	58
Active delirium/agitation	9 (7%)	63	22
Respiratory failure/airway compromise	33 (27%)	88	64
Epileptic seizure	4 (3%)	25	25
Cardiac arrest	5 (4%)	100	100
Procedural sedation	8 (7%)	88	13

mRS, modified Rankin Scale

^a^Data missing for seven patients

Forty-nine (40%) patients died or had their life support withdrawn during mechanical ventilation. Of these patients, two had an mRS score of 4, two had an mRS score of 5, and 45 were dead at 3 months. Thirty-one patients died in an ICU level unit, and 16 of these were treated as possible organ donors. The decision to withdraw life-supporting therapy was made at a mean of 3.8 ± 5.4 days after intubation.

One patient was moved to another hospital during mechanical ventilation, and one patient eventually remained ventilator dependent. In the remaining 70 patients who did not die in the ICU or after a withdrawal of life-sustaining treatment (WLST) decision was made, the duration of mechanical ventilation was 6.6 ± 7.2 days. Twelve (10%) patients required reintubation, and 18 (15%) underwent tracheostomy. ICU mortality was 26%. ICU length of stay was 7.7 ± 10.2 days in all patients and 8.5 ± 10.7 days in ICU survivors.

Forty-five (37%) patients died at the hospital. Of the survivors, three (4%) were discharged to home, 56 (74%) were discharged to rehabilitation, and 17 (22%) were discharged to other wards. A do-not-resuscitate order was placed on 78 (65%) patients, and 16 (13%) were treated as potential organ donors.

### One-Year Mortality and Functional Outcome at 3 Months

**Fig. 2:**
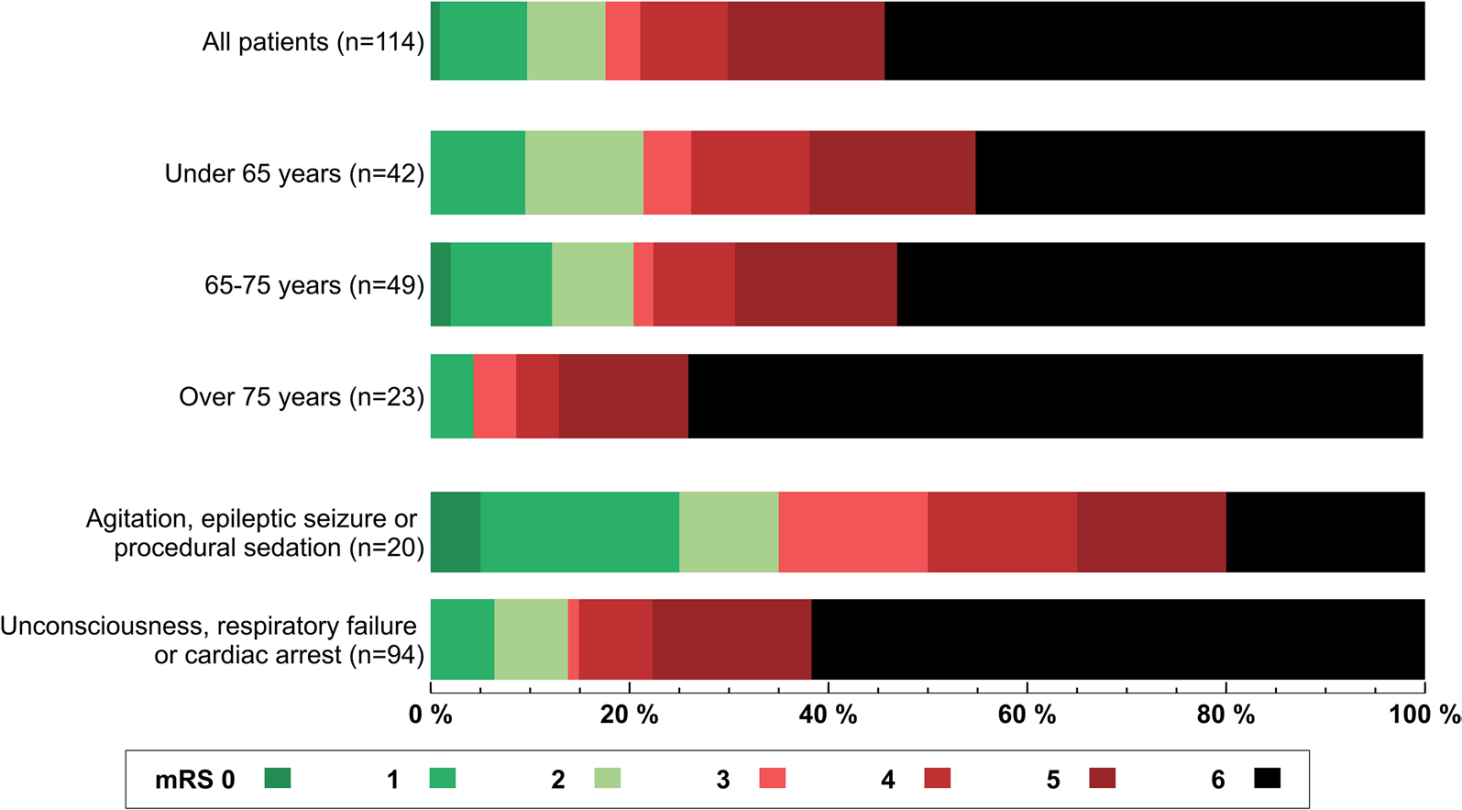
Functional outcomes of the patients. Subgroup results based on age and reason for intubation are shown as well. mRS, modified Rankin Scale

One-year mortality was 55%, and 82% had an mRS score of 3–6 at 3 months (Table 1, Fig. 2). Of the 66 one-year survivors, the residence of stay at 1 year was available for 47 (71%) patients. Of these, 34 (72%) patients were living at home with or without home care services and 13 (28%) were residing on a ward or in sheltered care.

Results of the logistic regression analyses for 1-year mortality are shown in Table 3. In the univariate analysis, older age and intubation due to unconsciousness, respiratory failure, or cardiac arrest were strongly associated with mortality. In the multivariate analysis, only age over 75 years and intubation due to unconsciousness, respiratory failure, or cardiac arrest remained significantly associated with mortality.

**Table 3:** Results of logistic regression analysis for 1-year mortality

Variables in the model	Mortality (%)	Univariate		Multivariate model with all indications for intubation		Multivariate model without preprocedural or cardiac arrest intubations	
		OR	95% CI	OR	95% CI	OR	95% CI
Age							
Under 65 years	43	1.00		1.00		1.00	
65–75 years	52	1.42	0.63–3.19	1.18	0.47–2.99	1.18	0.46–3.06
Over 75 years	80	5.26	1.67–16.58	4.88	1.25–19.13	4.99	1.23–20.30
Sex							
Male	55	1.00		1.00		1.00	
Female	55	1.00	0.48–2.10	0.70	0.29–1.71	0.60	0.24–1.54
CCI							
0	45	1.00		1.00		1.00	
At least 1	63	2.02	0.97–4.23	2.12	0.88–5.09	1.96	0.79–4.86
NIHSS at admission							
0–15	45	1.00		1.00		1.00	
16–42	64	2.13	1.01–4.49	1.41	0.60–3.33	1.35	0.55–3.29
Reason for intubation							
Agitation, epileptic seizure, or procedural sedation	19	1.00		1.00		1.00	
Unconsciousness, respiratory failure, or cardiac arrest	62	6.93	2.17–22.16	8.34	2.23–31.11	5.37	1.24–23.35
Intravenous thrombolysis and/or endovascular thrombectomy							
No	64	1.00		1.00		1.00	
Yes	49	0.54	0.25–1.17	0.57	0.23–1.41	0.59	0.23–1.49

Mortality and ORs with 95% CIs for 1-year mortality are shown. The multivariate models included age, CCI, NIHSS score at admission, reason for intubation, and acute stroke therapies (intravenous thrombolysis and/or endovascular thrombectomy)

CCI, Charlson Comorbidity Index, CI, confidence interval, NIHSS, National Institutes of Health Stroke Scale, OR, odds ratio

Three-month functional outcomes for all patients and subgroups are shown in Fig. 2. Three-month mRS scores were available for 114 (94%) patients, with the following distribution: 0–2, 18%; 3–5, 28%; and 6 (dead), 54%. In the univariate logistic regression analysis, intubation due to unconsciousness, cardiac arrest, or respiratory failure was associated with increased odds for an mRS score of 3–6 (OR 3.36, 95% CI 1.13–9.98). In the multivariate logistic regression analysis, none of the included variables were associated with functional outcome at 3 months (Supplemental Table 1).

Because patients intubated for sedation for thrombectomy probably have a much better outcome that patients intubated because of cardiac arrest, we performed an additional sensitivity analysis excluding these groups from the multivariate model. This did not significantly affect the results (Table 3).

**Fig. 3:**
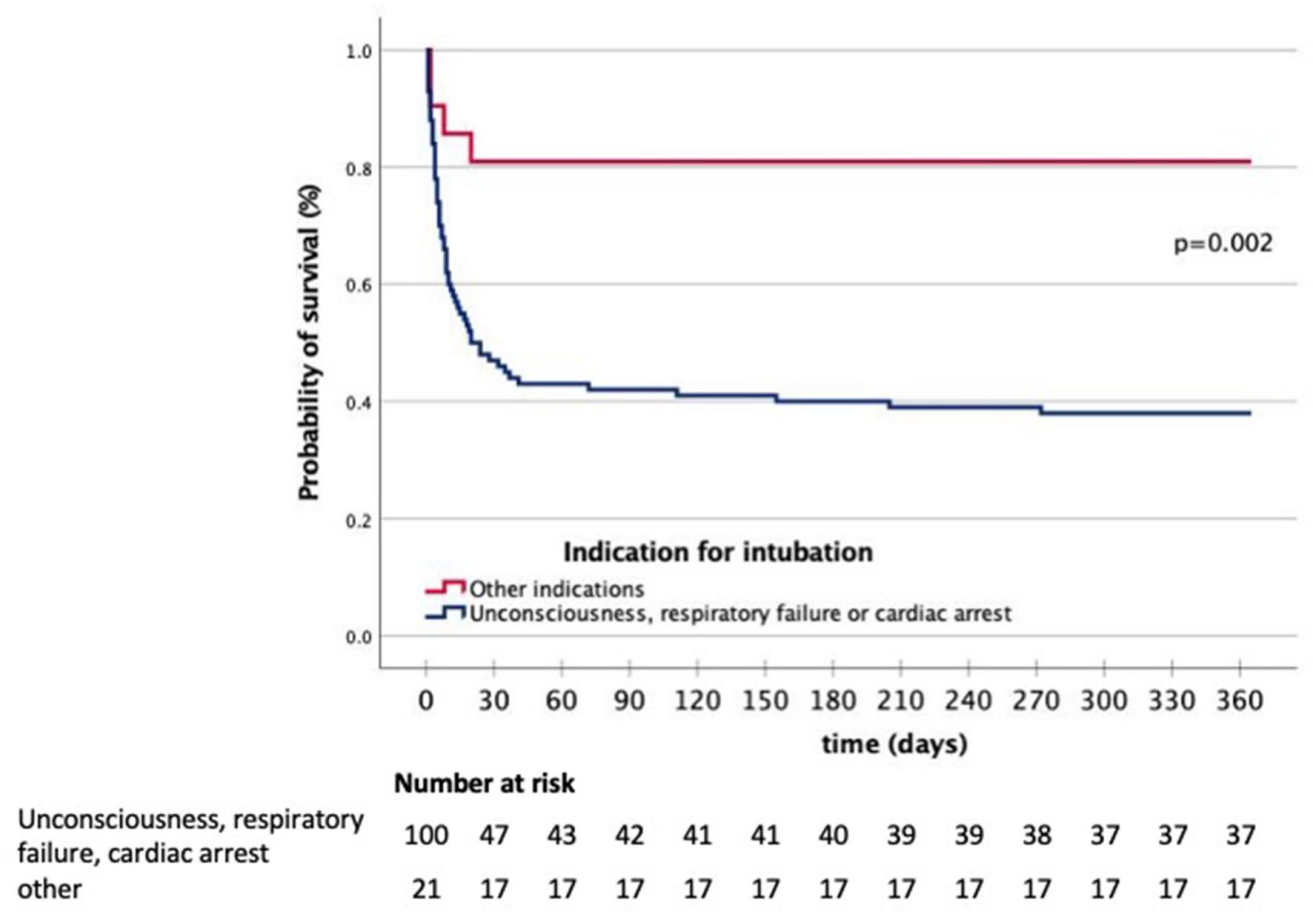
Survival of the patients stratified by the indication for intubation. Other indication: active delirium or agitation, hemodynamic instability, epileptic seizure, or preprocedural

The Kaplan–Meier curves for survival are shown in Fig. 3. Because mortality was similar in patients intubated for unconsciousness, respiratory failure, and cardiac arrest, these were combined as one subgroup. The rest of the patients formed the other subgroup. The mortality rate was highest in the first month after stroke (Fig. 3).

## Discussion

We studied the association of reason for intubation with 1-year survival in patients with acute ischemic stroke who required mechanical ventilation. We hypothesized that intubation due to a possibly reversible condition would be associated with better 1-year survival. In our retrospective study, 1-year mortality in patients with ischemic stroke who needed mechanical ventilation was 55%. In-hospital mortality was lower in our study (37%) than that in similar previous studies (53–57%) [4, 6–8]. In line with in-hospital mortality, 1-year mortality was also lower than that in previous studies (55% vs. 60–92%) [9–13]. In previous studies, most survivors were severely disabled, raising questions on the cost-effectiveness of ICU treatment for comatose patients with stroke [9, 31]. In our cohort, the proportion of patients with favorable 1-year outcome was significantly larger, as 72% of the 1-year survivors were able to return home. Of the 55 patients alive at 1 year, 36% had an mRS score of 0–2 at 3 months, reflecting the long recovery of the sickest stroke survivors.

Since 2015, mechanical thrombectomy has become standard in treatment of ischemic stroke caused by acute large vessel occlusions and has improved the prognosis substantially [28]. Because our cohort included patients admitted between 2016 and 2020, this change in treatment could explain why mortality was lower in our study than that in previous studies. Additionally, significant comorbidities and frailty were uncommon in our cohort, indicating that most patients had good prestroke functional status. This also could have contributed to the higher survival rates.

Our findings are in line with the prior reports that severely impaired consciousness is strongly associated with mortality among ventilated patients with stroke [9–11, 13, 31, 32]. Advanced age has also been associated with a poor prognosis in patients with stroke who need ICU treatment [5, 33]. Also in our study, age over 75 years was associated with mortality when adjusted for CCI, NIHSS score, acute stroke therapies, and the reason for intubation. The decision to intubate or to admit to the ICU is not based solely on age but more on the premorbid functional status and the predicted ability to recover from stroke.

The decision to intubate because of respiratory or airway failure warrants some discussion. Pneumonia is a common cause of respiratory failure and death after stroke. Dysphagia following stroke is associated with a worse functional outcome and increased mortality [34]. However, dysphagia following stroke is not always chronic, as some patients (particularly those with subcortical stroke) tend to only have a transient risk of aspiration [35]. In contrast, those with lesions in the brainstem, frontal operculum, or insular cortex tend to have more chronic problems with swallowing [35–37]. Some patients with stroke who require mechanical ventilation because of aspiration pneumonia may go on to have a satisfactory recovery because the pneumonia, as well as dysphagia, will resolve eventually.

In our study, however, intubation due to respiratory failure was associated with high mortality. Ischemic stroke may lead to disrupted breathing in many ways, notably either by leading to a reduced respiratory drive or causing bulbar weakness leading to aspiration [38]. The loss of protective airway reflexes and decrease in respiratory drive are often associated with the magnitude of neurological injury and level of consciousness. It is possible that many of the patients in the respiratory failure subgroup were then intubated because of neurological respiratory compromise and not a purely pulmonary problem. Sometimes respiratory failure is caused by heart failure, which is an independent predictor of unfavorable functional long-term outcome [39].

Of the 93 patients who were intubated because of unconsciousness, respiratory failure, or cardiac arrest, there were 15 patients who had an mRS score of 0–3 at 3 months. All these patients had some kind of a transient pathology leading to the need for mechanical ventilation. Seven patients had a basilar artery occlusion, which was recanalized. Two patients had a proximal occlusion of the middle cerebral artery, which was subsequently endovascularly recanalized. Four patients were intubated because of respiratory failure, of whom one had a pulmonary embolism, one had an acute severe angioedema, and two had a rapid atrial fibrillation leading to pulmonary edema. One patient had a cerebellar infarction that needed posterior fossa decompression. The last patient had a period of hypotension and simultaneous administration of opiates leading to prolonged loss of consciousness and ultimately intubation.

This study has some strengths. Helsinki University Hospital is a large comprehensive stroke center that treats all patients who are eligible for IV thrombolysis within an area of over one million inhabitants. It also serves as the sole center offering mechanical thrombectomy within a catchment area of 1.9 million inhabitants. Some key variables, such as NIHSS scores and 3-month mRS scores, were collected in a structured, prospective manner. We have no missing data regarding mortality, our primary outcome.

Our study also has limitations. First, most of our data are retrospective, collected from hospital records. There were no prespecified definitions for the different indications for intubation. The indications for intubation were gathered from hospital records, which sometimes lacked detailed information about the decision to intubate. There was some overlap in the reasons to intubate, but most of these were between agitation and periprocedural sedation or unconsciousness and respiratory failure.

Furthermore, only long-term vital status was available, and evaluation of long-term functional outcomes with an appropriate tool (i.e., mRS score) would have added value to the study. The mRS scores were not available for all patients from our prospective registry. However, 65.3% (79 of 121) of the patients had thrombolysis or endovascular treatment, and these patients’ 3-month mRS scores were assessed by an independent physician as part of the ongoing stroke quality registry in the study institution. It is also of note that the vital status of the patients was obtained from an official registry, limiting the bias in these patients.

The patients were treated in a neurologist-lead acute stroke care unit or in medical ICUs and not in a specialized neurocritical care unit, which may have affected the outcome. As in all studies focusing on populations with a high rate of withdrawing life-supporting therapy, our study bears an inherent bias of self-fulfilling prophecy [40]. It is possible that some patients who were deemed to have a pessimistic prognosis were not offered mechanical ventilation at all. The number of patients was relatively small, especially the number of patients intubated for reversible causes. Because of the retrospective setting, we cannot exclude the possibility that some of the patients might have lived if a withdrawal of life-sustaining therapy decision was not made.

Mortality in patients with acute ischemic stroke who require mechanical ventilation has remained high. The reason for intubation was strongly associated with the outcome, and some patients had a good outcome after ICU treatment. Selected patients with stroke may benefit from mechanical ventilation: when it is being used to treat a transient pathology, such as seizures or agitation, or as a part of general anesthesia for thrombectomy. It seems that invasive ventilation is often futile when prolonged unconsciousness or respiratory failure is caused by a truly irreversible neurological injury. Impaired consciousness and respiratory failure are often interconnected because both can worsen the other. The shift toward lower mortality should be addressed in future studies. The coming studies should focus on the treatment and outcome of the mechanically ventilated patients with stroke with possibly reversible conditions. Even though our results are not robust enough to guide individual treatment decisions, a treating physician might find these of use when discussing possible recovery trajectories with patients’ relatives.

### Supplementary Information

Below is the link to the electronic supplementary material.

Supplementary file1 (DOCX 32 kb)

## Data Availability

The statistical analyses and underlying data supporting the conclusions of this article will be made available by the authors to qualified researchers for research purposes, without undue reservation.
