# Supplementary material for: One-Year Survival of Ischemic Stroke Patients Requiring Mechanical Ventilation
Source: Neurocrit Care. 2023 Feb 9;39(2):348–56. doi: 10.1007/s12028-023-01674-9 (PMC10541824; doi:10.1007/s12028-023-01674-9)
Supplement: Supplementary file 1 — Supplementary file1 (DOCX 32 kb) [file 12028_2023_1674_MOESM1_ESM.docx]

## Supplemental Table 1

Results of logistic regression analysis unfavorable outcome (mRS 3-6) at 3 months. Proportion of patients with unfavorable outcome and odds ratios with 95 % confidence intervals are shown. The multivariate model included age, Charlson comorbidity index, NIHSS at admission and reason for intubation and acute stroke therapies (intravenous thrombolysis and/or endovascular thrombectomy).

| **Variable in the model** | | **Univariate** | | | **Multivariate** | |
| --- | --- | --- | --- | --- | --- | --- |
|  | |  |  |  |  |  |
|  | | **%** | **OR** | **95 % CI** | **OR** | **95 % CI** |
| Age | |  |  |  |  |  |
|  | Under 65 years | 79% | 1.00 |  | 1.00 |  |
|  | 65–75 years | 80% | 1.06 | 0.39-2.93 | 0.75 | 0.24-2.42 |
|  | Over 75 years | 96% | 6.00 | 0.71-50.76 | 4.61 | 0.47-45.04 |
| Sex | |  |  |  |  |  |
|  | Male | 86% | 1.00 |  | 1.00 |  |
|  | Female | 81% | 0.69 | 0.24-1.96 | 0.43 | 0.13-1.44 |
| CCI | |  |  |  |  |  |
|  | 0 | 75% | 1.00 |  | 1.00 |  |
|  | At least 1 | 89% | 2.61 | 0.92-7.38 | 2.22 | 0.70-7.10 |
| NIHSS at admission | |  |  |  |  |  |
|  | 0–15 | 75% | 1.00 |  | 1.00 |  |
|  | 16–42 | 89% | 2.67 | 0.96-7.41 | 1.80 | 0.59-5.54 |
| Reason for intubation | |  |  |  |  |  |
|  | Agitation, epileptic seizure or procedural sedation | 65% | 1.00 |  | 1.00 |  |
|  | Unconsciousness, respiratory failure or cardiac arrest | 86% | 3.36 | 1.13-9.98 | 3.06 | 0.86-10.83 |
| Intravenous thrombolysis and/or endovascular thrombectomy | |  |  |  |  |  |
|  | No | 90% | 1.00 |  | 1.00 |  |
|  | Yes | 78% | 0.39 | 0.12-1.24 | 0.32 | 0.08-1.27 |


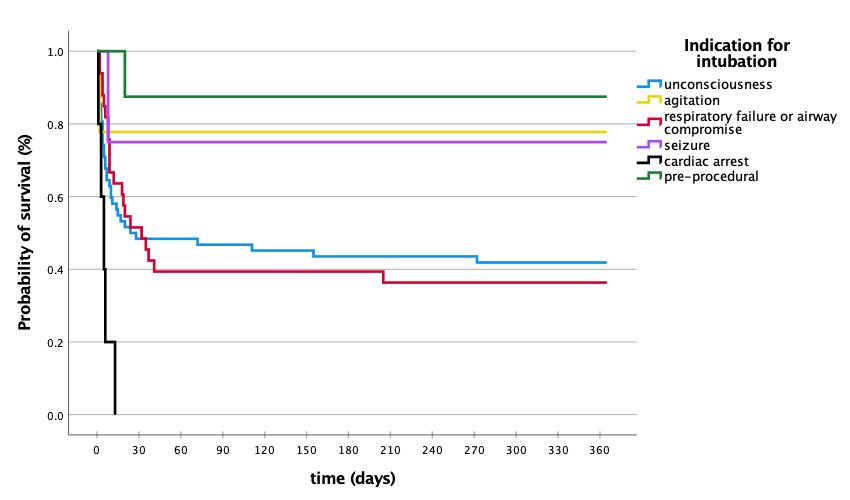


Supplemental Figure 1. Kaplan-Meier survival curve stratified by the indication for intubation.
